# Selected Polyphenols of Polish Poplar Propolis as a Key Component Shaping Its Antibacterial Properties—In Vitro and In Silico Approaches

**DOI:** 10.3390/molecules30092036

**Published:** 2025-05-03

**Authors:** Małgorzata Dżugan, Michał Miłek, Ewa Ciszkowicz, Andrzej Łyskowski, Monika Tomczyk

**Affiliations:** 1 Department of Chemistry and Food Toxicology, Institute of Food Technology and Nutrition, Faculty of Technology and Life Sciences, University of Rzeszów, Ćwiklińskiej 1a, 35-601 Rzeszow, Poland; mdzugan@ur.edu.pl (M.D.); mwesolowska@ur.edu.pl (M.T.); 2 Department of Biotechnology and Bioinformatics, Faculty of Chemistry, Rzeszów University of Technology, al. Powstańców Warszawy 6, 35-959 Rzeszow, Poland; ewa.ciszkowicz@prz.edu.pl (E.C.); alyskowski@prz.edu.pl (A.Ł.)

**Keywords:** propolis, antibacterial activity, human health, *p*-coumaric acid, galangin, pinocembrin, molecular docking

## Abstract

Propolis is a natural antibacterial medicine with a varied content of phenolic compounds, which determines the activity of the ethanol extract of propolis (EEP). A new attempt was made to standardize ethanol propolis extract via its conversion into a dry concentrate (dEEP) through a two-step process. Four samples of poplar propolis from the same geographical region were used for the study. Obtained dry concentrates reconstituted in 70% ethanol (500 μg/mL) were analyzed for their antioxidant properties, total phenolic and flavonoid contents, as well as HPLC polyphenol profile. It was shown that dEEP solutions in 70% ethanol, regardless of the diversified quality of the raw material, have equalized antioxidant properties and phenolic and flavonoid contents compared to raw EEPs. However, quantitative differences in the nine individual components were still found by HPLC-DAD. The antibacterial activity of the dEEP solutions (0.03–500 µg/mL) was compared with three individual polyphenols’ effect against Klebsiella pneumoniae and Streptococcus agalactiae. Based on the obtained MIC values and anti-biofilm activity of dEEPs compared to pure polyphenols, it was established that the effectiveness of the extract results from the combined action of flavonoids and phenolic acids. The antibacterial effectiveness of *p*-coumaric acid, galangin, and pinocembrin was additionally modeled using in silico analyses, suggesting promiscuous binding of all tested polyphenolic ligands to target enzymes.

## 1. Introduction

Propolis is a unique natural remedy, used since ancient times in traditional medicine, and its pharmacological properties are under continuous investigation [1,2,3]. Among bee products, it stands out specifically for its high antioxidant, anti-inflammatory, anticancer, and antimicrobial effects. Propolis, also known as bee glue, produced by Western honeybees from materials collected from plants, is used by bees in a hive as a building material that provides thermal insulation to seal the cracks in wooden walls and other parts of the hive and to strengthen the construction of wax combs; it is also used for hive disinfection [4,5].

**Figure 1:**
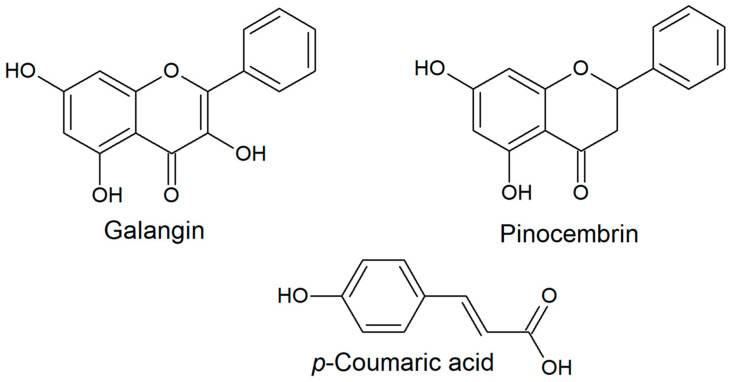
Selected propolis polyphenol structures. The structures were drawn using ChemSketch (v. 2017.2.1) software.

The composition of propolis is responsible for its biological properties, and the composition of poplar propolis is commonly as follows: about 50% comprises resinous substances, 30% beeswax, 10% volatile substances, 5% pollen, and 5% mechanical impurities [6,7]. However, a review of experimental data indicated that the average composition of propolis is somewhat different: more than 50% (even up to 70%) is made up of resinous substances, up to about 25% contains wax, and essential oil and aromatic components usually make up less than 1% [8]. Poplar propolis is rich in aromatic acids and their esters, the most important of which is caffeic acid phenylethyl ester (CAPE), which has a wide range of biological properties. Flavonoids, such as chrysin, apigenin, and galangin, are also important for the biological properties of propolis [9]. The content of flavonoid compounds in 10% ethanol extracts of Polish propolis samples from different regions of the country (*n* = 15) ranged from 6.2 to 18.8% [9]. Among the flavonoids, pinocembrin was found in the largest amount (average 4.7%), followed by pinobanksin (average 3.1%), galangin (average 2.2%), and chrysin (average 2.1%) [10]. On this basis, it can be assumed that poplar propolis is a rich source of pinocembrin—a compound belonging to the flavanone group [11]. On the other hand, our earlier study showed that galangin is a typical flavonol occurring in Polish propolis [12]. Five phenolic acids, namely gallic, caffeic, *p*-coumaric, ferulic, and *t*-cinnamic acid, have been identified in poplar propolis. In Polish samples, similarly to Romanian ones, a large amount of *p*-coumaric acid was detected (3452.608 and 3537.61 µg/g of dry extract, respectively) [13]. These bioactive molecules (Figure 1) have been studied individually and as ingredients of propolis extracts, and they can be used as representative markers for propolis standardization.

The bioactive molecule profile of raw propolis varies according to the geographical and botanical origin, season, bees’ genetics, and environmental factors, including beekeepers’ techniques and practices [14]. The appearance and physical properties of propolis also vary according to its geographical location and vegetation [15]. The most abundant propolis type is poplar propolis, with the plant source being *Populus* spp., mostly *P. nigra* L., which is abundant in flavones, flavanones, and phenolic acids and their esters [16]. Its chemical composition is diversified but completely different from Russian birch propolis and Brazilian green or red propolis. Propolis is not used raw; it must be processed into an extract. For commercial use, propolis extracts are often prepared with suitable solvents, such as ethanol, methanol, chloroform, ether, and acetone, of which ethanol extract is popular due to its high level of bioactivities [9,17]. It was determined that the biological activity (measured as antioxidant and antibacterial activity) of propolis extract is determined by both the quality of the raw material and the extraction method [18,19]. Meanwhile, the standardization of propolis extracts is a key factor influencing their efficacy [20].

It should be noted that to obtain products that can be marketed as functional foods or medicines, the same chemical composition must be established and standardized in order to adhere to the guidelines established in studies on safety and efficacy [5]. Additionally, standardization is necessary for batch-to-batch reproducibility in future productions. The selection of a biomarker for propolis extracts would not only depend on the therapeutic focus but also on other issues, such as reports regarding isolated compounds and reports matching the marker with the desired activity [21].

Currently, in the era of the growing threat of the multidrug resistance of microorganisms, scientists and the world are once again turning to natural medicine as a means of preventing and combating this threat [22]. As a result, in recent years, the specific antimicrobial properties of propolis have been rediscovered and studied. The antibacterial activity of propolis takes place due to its active compounds, such as aromatic compounds (caffeic acid) and flavonoids [23,24]. Propolis exhibits antibacterial effects through various mechanisms: the inhibition of bacterial cell division, the destruction of the cell wall and cytoplasm [23], and the stopping of protein biosynthesis [25,26]. 

Taking the above into account, in this work, research was undertaken on the standardization of dry extracts of poplar propolis and the evaluation of their antioxidant and antibacterial activities in vitro. Due to the fact that domestic Polish propolis is a rich source of pinocembrin, galangin, and *p*-coumaric acid, these three polyphenols were used for comparison during the in vitro study as well as in in silico simulations aiming to explain the mechanism of antibacterial action of propolis.

## 2. Results and Discussion

### 2.1. Polyphenol Content and Antioxidant Properties

Four poplar propolis samples originating from one geographical region were converted into dry concentrates (dEEP) by the two-step method with the yield of 100 g per 1 kg of raw material (10 ± 1%). This is an important observation as raw propolis samples differed regarding the content of impurities insoluble in 95% ethanol (51.6%, 43.6%, 77.7%, and 52.4% for samples 1, 2, 3, and 4, respectively). Each dEEP solution reconstituted in 70% ethanol (0.5 mg/mL) was assessed in terms of the antioxidant activity (FRAP, DPPH, and CUPRAC) and the total phenolic (TPC) and flavonoid (TFC) compound contents using spectrophotometric methods. All of the tested dry propolis extracts exhibited a slight but statistically different (*p* < 0.05) content of phenolic compounds, expressed as gallic acid equivalent (GAE), ranging from 163.10 to 188.10 mg GAE/g (Table 1). The flavonoid content (TFC) ranged from 62.60 to 86.41 mg QE/g, and the significantly lowest value was observed for dEEP2 (*p* < 0.05). The calculated TFC-to-TPC ratio was similar (*p* > 0.05), excluding sample dEEP2, and was within the previously reported range [27]. Similarly, Kurek-Górecka et al. [13] determined that the phenolic concentration in Polish poplar propolis dry extracts was 123 mg GAE/g, while for Romanian samples, the concentration of polyphenols ranged between 123.92 and 155.28 mg GAE/g.

Our previous studies showed a high correlation between the activity of the ethanol extract of EEP and the content of impurities insoluble in 95% ethanol. Depending on the purity of the raw propolis, the phenolic content ranged from 32.2 to 90 mg GAE per g of raw propolis [28]. In a study conducted by Marquele et al. [29], it was shown that the total phenolic content ranged from 37.83 to 46.02 mg GAE/g in dry propolis extracts obtained by concentration in a rotating evaporator and by spray-drying of Brazilian green propolis commercial ethanolic extracts. This indicates that the difference in phenolic compounds is due not only to the botanical and geographical origins but also depends on the solvent and drying method used.

The antioxidant activity of the analyzed extracts assessed based on reducing properties towards Fe^3+^ ions (FRAP) and Cu^2+^ ions (CUPRAC), as well as the hydrogen-donating ability (DPPH), was relatively balanced in all analyzed samples (Table 2). The antioxidant activity of the extracts showed a variability from 3.31% (FRAP) to 5.01% (DPPH) and was 10-fold lower than that observed for raw ethanol extracts of propolis of the same origin, which was within the range of 30.41% (DPPH) to 35.23% (FRAP) [28]. Regardless of the method used, the lowest activity was observed in sample dEEP1, and the highest in sample dEEP3, and statistically significant differences confirming this difference were found only in the case of the DPPH test (*p* < 0.05).

The correlation analysis between the determined parameters suggests that the high content of phenolic compounds strongly affects the antioxidant activity of propolis extract, which was especially observed in the FRAP and DPPH methods (r = 0.648 and 0.875, respectively). The total flavonoid content was significantly (*p* < 0.05) correlated with antiradical activity (DPPH, r = 0.959). In turn, the CUPRAC method showed smaller differences between the extracts, which may be due to the presence of specific compounds with the effect of reducing Cu^2+^ ions in these extracts. A weaker correlation between the CUPRAC results and the TPC (r = 0.146) and TFC (r = 0.564) was found. The full correlation matrix is shown in Table S1.

As the antioxidant properties of propolis are largely attributed to galangin and pinocembrin, which, thanks to the number and arrangement of hydroxyl groups, have the ability to neutralize free radicals, these flavonoids were tested with the use of standard colorimetric tests, and *p*-coumaric acid was chosen as a representative phenolic acid. The mechanism of antioxidant activity of tested components involves the donation of hydrogen atoms or electrons, which leads to the stabilization of reactive oxygen species and delays oxidative processes [24]. Moreover, the reduction or chelation of metal ions is also possible [6]. Among tested polyphenols, galangin shows the highest antioxidant activity in all methods, indicating strong reducing properties and the ability to neutralize free radicals. Due to the presence of three hydroxyl groups, galangin can easily donate a hydrogen atom, especially from the OH group at position 3 [30]. Numerous studies have shown its protective potential against oxidative stress, especially through the activation of antioxidant enzymes such as superoxide dismutase, catalase, and glutathione S-transferase [30]. Quantum mechanical studies have shown that, compared to other flavonoids, galangin is a moderate antioxidant; however, it is effective in reducing and chelating copper(II) ions [31]. Surprisingly, pinocembrin exhibited the lowest activity in each of the used tests, suggesting its limited antioxidant potential. This confirms the results of theoretical studies, which indicate that pinocembrin has weak antiradical activity and moderate metal ion chelating activity [32]. In turn, *p*-coumaric acid shows particularly high activity in CUPRAC, indicating that it can reduce copper ions (Cu^2+^ → Cu^+^) better than other compounds. However, its ability to neutralize free radicals (DPPH) and reduce Fe^3+^ (FRAP) is lower than that of galangin. In vitro studies of the action of *p*-coumaric acid as an antioxidant, using four methods, namely DPPH, ABTS, FRAP, and metal ion chelation, showed that this compound has a dose-dependent antioxidant potential involving various mechanisms; however, its antioxidant strength was smaller than that of the control substances used (Trolox and EDTA) at analogous concentrations [33]. However, at the same time, its potential as an antioxidant compound in the treatment of hyperlipidemia has been demonstrated in an in vivo study [33].

### 2.2. Polyphenolic Profile

The pharmacological properties of propolis usually result from the presence of individual phenolic compounds [4,24,34]. Previous studies confirmed the differentiation of antioxidant activity of the tested propolis samples [28], while the proposed method of obtaining a dry extract equalizes the content of the tested bioactive compounds. Similar observations were noted by Özkök et al. [35], who assessed the total content of polyphenols and flavonoids in propolis extracts obtained using different solvents. In their study, it was revealed that propolis, which differs in botanical origin and bioactive component content, can be prepared in a specific way depending on the type of solvent and concentration. Due to the small amount of studies available on this subject, the obtained results may be useful for the basic standardization of propolis.

The dEEPs reconstituted in 70% ethanol (10 mg/mL) were analyzed by HPLC-DAD. All analyzed samples were characterized by a similar phytochemical profile. The dominant peaks corresponded to *p*-coumaric, ferulic, and benzoic acids. Caffeic acid and its phenylethyl ester (CAPE), vanillin, and flavonoids, including pinobanksin, pinocembrin, and galangin, were also identified but in smaller amounts. In addition to the identified compounds, there were other minor acids and flavonoids present in the extracts that were not identified due to the lack of standards.

The contents of identified phytochemicals in the tested extracts are shown in Table 3.

The most abundant fraction was phenolic acids, with *p*-coumaric acid being the dominant one, and its content ranged from 24.86 to 46.70 mg/g of dry extract. A significantly lower content of determined phenolic acids was shown by extract dEEP1, which corresponded to the lowest content of total phenols (Table 1). However, the contents of flavonoids and CAPE were the highest in this extract. Sample EEP3 had the lowest contents of flavonoids, vanillin, and CAPE but contained more phenolic acids.

In our previous studies on propolis from the same region, the highest contents were also recorded for flavonoids, including chrysin, pinocembrin, and pinobanksin [12,28]. However, the polyphenol profile differs when determined in the raw, liquid ethanol extract of propolis and the propolis concentrate obtained after the evaporation of ethanol and freeze-drying of the residue. The fraction subjected to lyophilization mainly contained compounds of higher polarity, such as phenolic acids, similar to aqueous extracts of propolis [19]. It is known that flavonoids are particularly sensitive to temperature; during heat treatment, they may degrade or convert into various derivatives [36]. In turn, phenolic acids showed relatively high thermal stability, and complete decomposition was observed at temperatures of 300–350 °C [37]. Although freeze-drying is considered a technique for preserving natural products that only slightly change the phytochemical composition of the products, it is known that the losses are the smallest for condensed tannins, while for other polyphenols, the effect may be different and undesirable [38].

Similar contents of phenolic acids in freeze-dried extracts of propolis from different regions of Turkey were observed by Izol et al. [39]. The content of caffeic acid in this study ranged between 6 and 47.78 mg/g, while that of *p*-coumaric acid ranged between 4.80 and 66.45 mg/g [39]. The content of individual polyphenols was also tested for extracts from different regions of the world, including the Polish propolis extract, which contained caffeic acid (0.42 mg/g), *p*-coumaric acid (3.45 mg/g), and ferulic acid (0.31 mg/g) [13]. The content of pinocembrin according to the cited study was only 0.0088 mg/g, and that of CAPE was 0.35 mg/g, while in our study, the contents were 0.27–4.21 and 0.90–6.42 mg/g, respectively. Other samples of extracts prepared from propolis from Romania, Turkey, and Uruguay had a similar composition [13]. Galangin (0.92–4.16 mg/g) was determined in all tested extracts, which was previously considered a characteristic component of propolis from Poland [12]. This flavonoid has also been recognized as one of the markers enabling the determination of the geographical origin of propolis from different regions of Italy [40].

**Figure 2:**
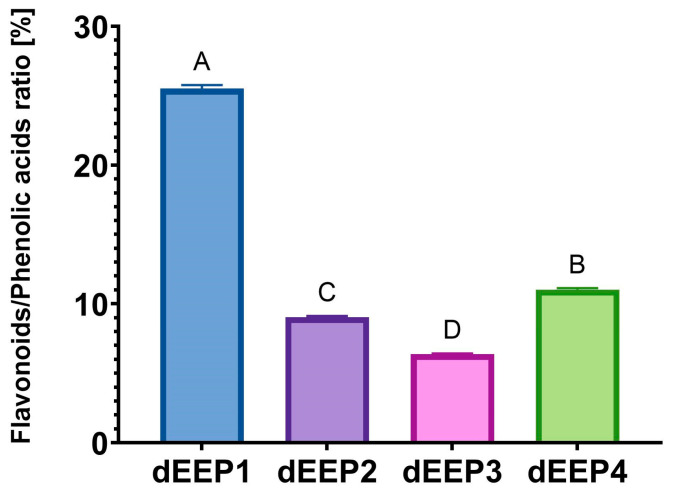
The ratio of flavonoids to phenolic acids calculated based on HPLC-DAD results. The data are shown as means ± SD. ^A,B,C,D^—mean values sharing the same superscript letter are not significantly different (*p* > 0.05).

As an additional indicator, the ratio of the summed content of flavonoids to phenolic acids (including CAPE) was determined (Figure 2). We previously proposed such an indicator as a determinant of the bioactivity of propolis extracts [27]. However, a statistical analysis did not confirm the occurrence of significant correlations between the proposed indicators and bioactivity (Table S1). Nevertheless, the role of flavonoids in shaping the biological activity of propolis is widely considered, and the total flavonoid content has been proposed as an indicator for the standardization of extracts [41].

Among the four extracts analyzed, the highest percentage of flavonoids in relation to phenolic acids was found for dEEP1 with a value of over 25%. For the previously analyzed propolis extracts, this value ranged from 40 to 176%, with extremely high contents resulting from the particularly high content of chrysin in two samples [27]. However, it should be remembered that not all flavonoids were quantitatively analyzed in the current study, and some of them remain unidentified. On the other hand, in the cited earlier work, CAPE was not identified and quantified. This has an obvious impact on the flavonoid/phenolic acid ratio.

### 2.3. Antibacterial Properties

The assessment of antibacterial activity is essential for evaluating the therapeutic potential of natural extracts. In this study, the minimum inhibitory concentration (MIC) values and anti-biofilm properties were determined to characterize the effects of propolis extracts and standards (galangin, pinocembrin, and *p*-coumaric acid) against *K. pneumoniae* and *S. agalactiae*. The MIC and anti-biofilm assay indicate, respectively, the lowest extract concentration that inhibits bacterial growth and the ability of the extracts to prevent biofilm formation [42]. These complementary methods provide comprehensive insights into the antimicrobial properties of the tested extracts. It was observed that *S. agalactiae* is more sensitive to all tested extracts with the lowest MIC values of 40.00 µg/mL (dEEP3 and dEEP4) and 16.00 µg/mL (pinocembrin) (Table 4). According to the pure polyphenols, both bacterial strains were resistant to galangin, and *K. pneumoniae* was also resistant to p-coumaric acid. *K. pneumoniae* exhibited resistance to oxacillin, a narrow-spectrum penicillin [43], even at the highest tested concentration (250 µg/mL). This finding aligns with results reported by other researchers who observed MIC values exceeding 250 µg/mL [44]. In contrast, our results demonstrate that *K. pneumoniae* was susceptible to dEEP1 and dEEP2 (MIC = 625.00 µg/mL) and pinocembrin (MIC = 32.00 µg/mL). Chloramphenicol and streptomycin displayed considerably higher antibacterial activity, with MIC values of 7.81 µg/mL and 1.95 µg/mL, respectively, which is also consistent with the findings of other authors [45]. However, considering that besides its antibacterial activity, propolis extract has other beneficial effects, e.g., antioxidant and anti-inflammatory effects, and that it is safer for the intestinal microbiota [12], it seems to be a better alternative to classic antibiotics.

**Figure 3:**
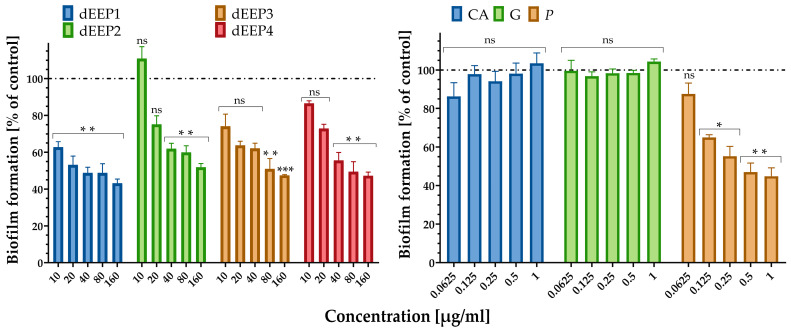
Anti-biofilm activity of dEEP1–dEEP4 (**left**) and standards (CA—*p*-coumaric acid; G—galangin; and P—pinocembrin) against reference *S. agalactiae* strain. Statistical significance between groups treated with different concentrations of AMPEC4 and non-treated control (* *p* < 0.05, ** *p* < 0.01, and *** *p* < 0.001) (**right**) calculated with Dunnett’s multiple comparisons test (GraphPad Prism 8.1, Boston, MA, USA).

*Streptococcus agalactiae*, in rare cases, can be found in the oropharynx and may contribute to throat infections, particularly in individuals with weakened immune systems. If untreated, strep throat can cause complications, such as kidney inflammation or rheumatic fever [46]. Our study demonstrates that propolis extracts, especially dEEP1, which allow for the formation of only approximately 60% of the biofilm compared to the untreated control, exhibit antimicrobial properties, including the ability to inhibit biofilm formation by *Streptococcus agalactiae*. Since biofilms protect bacteria from external threats, including antibiotics and the host immune response, preventing their formation can make the bacteria more susceptible to antimicrobial agents and immune defenses. This suggests that propolis may reduce the viability of *S. agalactiae* and potentially aid in controlling infections caused by this bacterium, including its presence in the throat. The highest anti-biofilm activity was observed for dEEP1 with 37%, 47%, and 57% inhibition in its formation after incubation with 10, 20, and 160 µg/mL, respectively. Other dEEPs in a concentration of 160 µg/mL, dEEP2, dEEP3, and dEEP4, inhibited biofilm formation by 48%, 52.5%, and 52.7%, respectively, thus exhibiting lower anti-biofilm properties (Figure 3).

The highest overall antibacterial activity of dEEP1, manifested by the lowest MIC value and *S. agalactiae* biofilm formation, may be related to the highest pinocembrin content (4.21 mg/g), whose antibacterial properties were also observed earlier against oral bacteria [47]. This may be supported by the data from the HPLC-DAD analysis (Table 3), which revealed that 160 µg of the dEEP1 fraction contained 0.67 µg of pinocembrin. An analogous amount of pure pinocembrin caused a 65% inhibition of biofilm formation, which can be compared to the total dEEP1 concentration, which exhibited a 67% inhibition. Additionally, neither pure *p*-coumaric acid nor galangin, other components of dEEP, exhibited anti-biofilm activity against *S. agalactiae*.

### 2.4. Bioinformatic Analysis of Putative Virulence Factors

In order to identify putative virulence factors related to reference bacterial strains, UniProt database [48] resources were used. The following reference proteomes were found and selected: *Streptococcus agalactiae* serotype V (strain ATCC BAA-611/2603 V/R) and *Klebsiella pneumoniae* subsp. *pneumoniae* (strain HS11286).

For each of the proteomes, secreted cellular components (proteins) not associated with the cell wall were selected for further analysis. The proteins were selected based on the keyword search (keywords: cellular component; secreted) and built-in UniProt filters. Six proteins and one protein satisfying the above criteria were found for the investigated strains *S. agalactiae* and *K. pneumoniae*, respectively. Table 5 lists the respective UniProt codes, reported protein function, the link to the UniProt resources, and the availability of the AlphaFold model.

**Figure 4:**
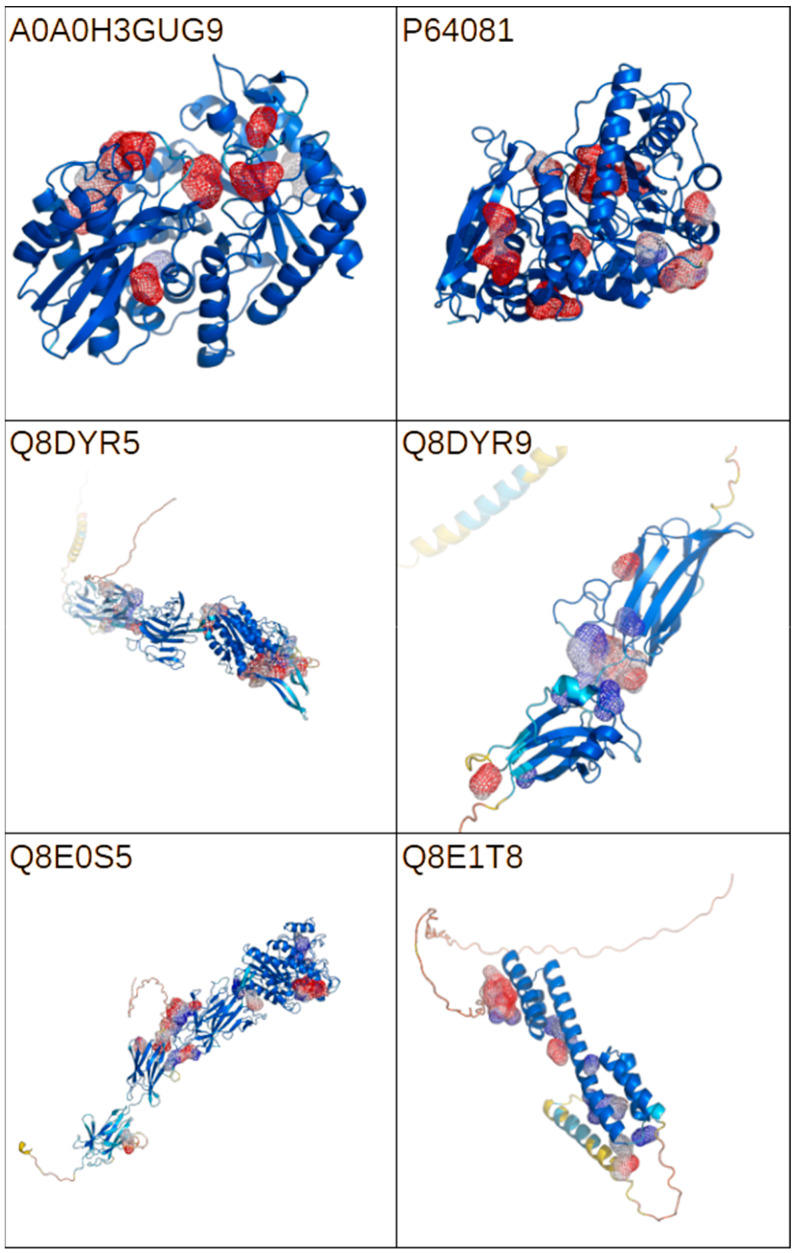
A graphical representation of the AlphaFold models for the selected proteins represented by the UniProt codes. The color scheme represents model confidence, with red, yellow, and blue representing values ranging from low to high according to the pLDDT score. Cavity detection was performed using the CavitOmiX (v. 1.0) plug-in for the molecular visualization system PyMOL (open source, v. 3.1.0). The top 10 cavities are represented by mesh colored according to the Coulomb potential. The figure was prepared with PyMol opensource version 3.1.0.

AlphaFold computational structural models are available for six of the seven investigated proteins. Serine protease Q8DX06 lacks both experimental and computational structural information. For each of the proteins, a cavity analysis was performed. The results of the analysis are presented in Figure 4. The identification of all potential binding sites would allow for the evaluation of the interactions between model compounds and the molecular targets—potential bacterial virulence factors. The performed analysis identified numerous cavities with varied volumes, suggesting the availability of multiple binding for a wide range of potential ligands.

**Figure 5:**
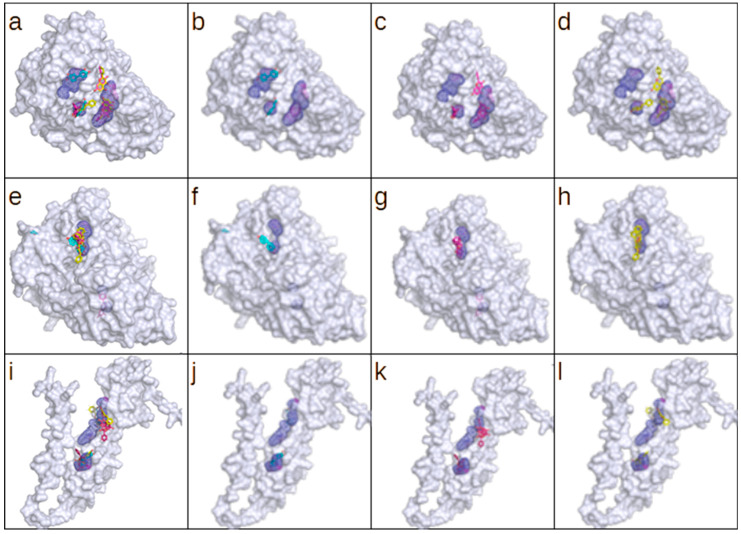
The results of the docking simulation for (**a**–**d**) A0A0H3GUG9, (**e**–**h**) P64081, and (**i**–**l**) Q8E1T8. Panels (**a**,**e**,**i**) present the combined top three scoring poses for each tested ligand: galangin (blue), pinocembrin (magenta), and *p*-coumaric acid (yellow). Panels (**b**–**d**,**f**–**h**,**j**–**l**) present the top-scoring poses for each ligand separately. Cavity detection was performed using the CavitOmiX (v. 1.0) plug-in for the molecular visualization system PyMol. Cavities matching the predicted ligand position are shown as mesh. The figure was generated using PyMOL (open source, v. 3.1.0).

Out of seven putative virulence factors with available structural models, three have assigned predicted enzymatic function (enolases: P64081 and A0A0H3GUG9; peptidase Q8E1T8). They were selected for a docking simulation with the following substrates: galangin (PubChem ID: 5281616), pinocembrin (PubChem ID: 68071), and *p*-coumaric acid (PubChem ID: 637542). A blind docking simulation was performed with the server DockThor (https://dockthor.lncc.br/, accessed on 25 March 2025). The results for each tested protein are tabularized in Table 6. A visualization of the results, including the top three scoring poses for each ligand, are presented in Figure 5. The calculated energy scores are comparable for galangin and pinocembrin irrespective of the molecular target. The conformity of the obtained scores can be attributed to the high degree of structural similarity and results in similar geometrical compatibility with available cavities. That also explains the much lower energy scores obtained for *p*-coumaric acid, which is significantly smaller and more flexible. This property is visible in the partial energy score presented in Table 6. However, the total score calculated representing predicted binding affinity remains at the comparable level for all tested ligands and targets. Smaller ligands with higher flexibility will have much greater chances to bind to accessible cavities. A graphical representation of the obtained docking poses confirms that both large ligands (galangin and pinocembrin) associate with the same cavities, and *p*-coumaric acid (small ligand) selects other binding sites. A noteworthy exception is present in the results obtained for P64081, where all three ligands bind in the vicinity of the same cavity.

The scope of the performed in silico analysis was hindered by a number of factors primarily related to the availability of suitable data. The available proteomes contained a low number of the annotated proteins, which could be assigned as putative bacterial virulence factors. However, we were able to show that the investigated molecular target surfaces are characterized by a number of cavities varying in size and representing putative binding sites for polyphenol components of the propolis. In all tested cases, the obtained energy scores are comparable, suggesting promiscuous binding for all tested ligands rather than one specific interaction. One notable exception is visible in the results obtained for enolase P64081, where all three ligands interact with a single specific cavity. Such well-defined results warrant further investigation into the role of that specific cavity in the biological activity of this enolase. The obtained in silico simulation results strongly suggest that the antibacterial activity observed for the ligands should be attributed to a combined effect represented by multiple protein–ligand interactions most likely interfering with the enzymatic activity of the putative virulence factors. However, the presented theoretical considerations have not been experimentally verified, which could confirm their validity.

The binding of polyphenolic compounds to protein targets is, however, just one of the possible mechanisms of the antimicrobial activity of propolis extract components. Studies of the action of pinocembrin against the bacterium *Aeromonas hydrophila* have shown that the flavonoid causes the degradation of the bacterial cell wall and, consequently, the leakage of key components of the bacterial cell [49]. For *Campylobacter* bacteria, the effect of pinocembrin on the increased permeability of cell membranes, and the decreased expression of certain proteins, including ribosomal proteins and those involved in iron uptake, has been demonstrated. This resulted in the modulation of bacterial metabolism and the disruption of protein biosynthesis and iron metabolism in cells [50]. For galangin, apart from bacterial cell wall damage, the inhibition of biofilm formation and reduction in the content of biomolecules in the cells of *Enterococcus faecium* bacteria have also been shown [51]. Cell membrane damage caused by galangin has also been observed in the treatment of *Staphylococcus aureus* [52]. *p*-Coumaric acid similarly affects the bacterial cell wall and can also bind to bacterial DNA, which might affect replication, transcription, and expression [53]. Moreover, this compound also caused a decrease in intracellular ATP, the hyperpolarization of a cell membrane, the degradation of whole cell protein, and the malformation of cell morphology when applied against *Alicyclobacillus acidoterrestris* [54]. The above shows how multifaceted the antibacterial effect of propolis components is, which should be emphasized in molecular studies concerning individual components, and the interactions between them are not considered in a complex matrix.

## 3. Materials and Methods

### 3.1. Chemicals and Reagents

Ethanol (puriss. p.a., 96%) and acetonitrile (HPLC grade, ≥99.9%) were purchased from Honeywell (Morristown, NJ, USA). Aluminum chloride, sodium carbonate, iron(III) chloride, copper(II) chloride, and ammonium acetate were purchased from Chempur (Piekary Śląskie, Poland). Folin–Ciocalteu reagent, 2,2-Diphenyl-1-picrylhydrazyl (DPPH), 2,4,6-Tris(2-pyridyl)-s-triazine (TPTZ), neocuproine, Mueller–Hinton Broth (MHB), Mueller–Hinton Agar (MHA), 1-(4,5-Dimethylthiazol-2-yl)-3,5-diphenylformazan (MTT), chloramphenicol, oxacillin, streptomycin, *p*-coumaric acid, caffeic acid, benzoic acid, ferulic acid, caffeic acid phenetyl ester (CAPE), vanillin, galangin, pinobanksin, and pinocembrin were purchased from Sigma Aldrich (Saint Louis, MO, USA).

### 3.2. Ethanolic Extracts of Propolis Preparation

Propolis samples (n = 4) used to prepare the extracts came from four apiaries in the Podkarpackie Province (southeastern Poland). Samples differed in terms of insoluble matter content (50–60%). Ten grams of each previously frozen and crushed propolis was mixed with 100 mL of 70% aqueous ethanol, shaken for 30 min (400 rpm, OrbiShaker MP, Benchmark, Tempe, AZ, USA), and then left to macerate for 5 days with occasional shaking. After this time, the extracts were filtered through filter paper and concentrated, with the ethanol being removed in a rotary evaporator (RVC 2–18 CDPlus, Martin Christ, Osterode am Harz, Germany). The concentrated residue was frozen and freeze-dried (Alpha 1–2 LD plus, Martin Christ, Osterode am Harz, Germany) to obtain four dry ethanol extracts of propolis (dEEP1–dEEP4).

### 3.3. Total Phenolic and Flavonoid Contents and Antioxidant Capacity

The total phenolic content was determined using Folin-Ciocalteu reagent, the total flavonoid content was determined using the AlCl_3_ method, and the antioxidant capacity was determined using the DPPH, FRAP, and CUPRAC methods as previously described [19]. Solutions of dry propolis extracts as well as pure compounds in 70% aqueous ethanol were used at a concentration of 500 μg/mL.

### 3.4. HPLC-DAD Analysis

For HPLC-DAD analyses, solutions of dry propolis extracts in 70% aqueous ethanol at a concentration of 10 mg/mL were filtered through nylon filters (0.22 μm) for further use. The apparatus and separation conditions were used as previously described [12]. The results were expressed in mg of individual compounds per g of dry extract using calibration curves prepared in the range of 12.5–250 μg/mL for caffeic acid, *p*-coumaric acid, ferulic acid, benzoic acid, vanillin, CAPE, pinobanksin, pinocembrin, and galangin (R^2^ > 0.997).

### 3.5. Antibacterial Activity

Antimicrobial activity and biofilm inhibition assays were conducted using certified bacterial cultures deposited by the Department of Biotechnology and Bioinformatics, Faculty of Chemistry, Rzeszow University of Technology (*Klebsiella pneumoniae* ATCC 13883 and *Streptococcus agalactiae* DSM 2134). Stock solutions of dry propolis extracts (5000 µg/mL) and pure phenolic compounds (500 μg/mL) in 70% aqueous ethanol were used.

#### Minimum Inhibitory and Anti-Biofilm Activity

The antibacterial efficacy of tested extracts (EEP1–EEP4) and standards (*p*-coumaric acid, galangin, and pinocembrin) was determined by establishing the minimum inhibitory concentration (MIC) and anti-biofilm activity in concentrations between 0.15 and 5000 µg/mL (for propolis extracts) and between 0.03 and 500 µg/mL (for standards) following the methodology previously described in [55,56,57]. The antibiotic susceptibility of each bacterial strain to chloramphenicol, oxacillin, and streptomycin was also evaluated in the concentration range of 0.02 to 250 µg/mL. The MIC was defined as the minimal concentration of the antimicrobial agent that completely inhibited visible microbial growth. The serial microdilution method was employed in Mueller–Hinton Broth to generate a range of concentrations of the tested extracts and standards. Only dilutions containing less than 12.5% aqueous ethanol were included in the analysis. The data represent the average of at least three independent biological replicates. All experiments were performed in triplicate. Positive (bacterial growth) and negative (media sterility) controls were included in each experiment. All reagents and bacterial cultures were prepared under aseptic conditions within an ESCO Airstream Laminar Flow Cabinet.

### 3.6. Structure Analysis and Visualization

Models of the investigated molecular targets were obtained either from the PDB database [58] (https://www.ebi.ac.uk/pdbe/; access date: 25 March 2025) or AlphaFold database [59] (https://alphafold.ebi.ac.uk/; access date: 25 March 2025).

Figures were prepared with PyMOL open source version 3.1.0 [60]. Cavities were calculated using CavitOmiX (v. 1.0, 2022, Innophore GmbH, Graz, Austria) [61,62]. The corresponding hydrophobicity module of the program VASCo was used to analyze the hydrophobicity of the cavities. The cavities were calculated using a modified LIGSITE algorithm.

### 3.7. Docking Simulations

Simulations were performed with DockThor web server [63] for protein ligand docking. Ligand files were obtained from PubChem (https://pubchem.ncbi.nlm.nih.gov/; access date: 25 March 2025). Corresponding codes for galangin, pinocembrin, and *p*-coumaric acid are reported in the manuscript text.

Docking was performed with default simulation parameters. Results for the top-scoring poses are reported.

### 3.8. Statistical Analysis

All analyses were performed in triplicate unless otherwise indicated. The results were presented as the mean ± the standard deviation (SD). Statistically significant differences between the particular samples for individual parameters were determined using a one-way ANOVA (*p* < 0.05), followed by Tukey’s test. To assess the relationships between the antioxidant activity parameters (FRAP, DPPH, TPC, TFC, and ORAC), Spearman’s rank correlations were calculated. Statistical analyses were conducted using Statistica 13.3 software (StatSoft, Inc., Tulsa, OK, USA), and graphs were generated in GraphPad Prism 10 Software (GraphPad Software, Boston, MA, USA).

## 4. Conclusions

The two-step vacuum evaporation/lyophilization method allows for crude ethanolic propolis extract to be converted into dry extract, which can be considered as the first step towards standardization. Based on the conducted colorimetric tests and HPLC polyphenol profile analysis, it can be stated that such conversion allows for compositional variability to be reduced. However, the use of the proposed indicators (TFC/TPC or flavonoids/phenolic acids) did not allow for the prediction of the bioactivity of propolis, which must be verified in biological tests. Testing antibacterial activity in vitro confirmed the net effect of the whole propolis extract results from various interactions between components (both flavonoids and phenolic acids) and is difficult to predict on the basis of results obtained for single compounds.

Molecular docking confirmed the importance of complex interactions between individual EEP components responsible for antibacterial activity, which can specifically or non-specifically bind to various virulence factors of pathogens. The complementary in vitro and in silico approaches confirmed the usefulness of bioinformatics in predicting and explaining the mechanisms of bioactivity of components of propolis; furthermore, they can allow for the most suitable microorganisms to be selected for the microbiological standardization of propolis extracts, which justifies continued research.

## Figures and Tables

**Table 1:** Total phenolic compounds (TPC) and flavonoids (TFC) as well as TFC-to-TPC ratio in individual samples of dry propolis extracts.

Sample	TPC [mg GAE/g]	TFC [mg QE/g]	TFC/TPC [%]
dEEP1	171.13 ± 4.97 ^bc^	86.41 ± 3.23 ^a^	50.55 ± 3.18 ^a^
dEEP2	163.10 ± 8.81 ^c^	62.60 ± 2.44 ^b^	38.42 ± 1.68 ^b^
dEEP3	179.56 ± 7.82 ^ab^	82.44 ± 3.90 ^a^	45.91 ± 0.63 ^a^
dEEP4	188.10 ± 6.15 ^a^	84.48 ± 5.3 ^a^	44.91 ± 2.38 ^a^

The data are shown as means ± SD. ^a,b,c^—mean values sharing the same superscript letter are not significantly different.

**Table 2:** Antioxidant activity of individual samples of dry propolis extracts and selected pure phenolic standards.

Sample	FRAP [μmol TE/g]	DPPH [μmol TE/g]	CUPRAC [μmol TE/g]
dEEP1	587.72 ± 3.31 ^a^	331.43 ± 20.94 ^a^	4325.64 ± 174.45 ^a^
dEEP2	590.35 ± 16.24 ^a^	288.77 ± 4.10 ^b^	4146.15 ± 160.62 ^a^
dEEP3	668.86 ± 31.10 ^b^	343.94 ± 16.66 ^a^	4412.82 ± 158.08 ^a^
dEEP4	627.19 ± 31.31 ^ab^	329.38 ± 23.16 ^a^	4169.23 ± 104.06 ^a^
**Phenolic standards**			
Galangin (500 μg/mL)	738.16 ± 8.00	338.20 ± 13.87	5887.18 ± 242.81
Pinocembrin (500 μg/mL)	6.80 ± 1.00	21.94 ± 6.16	1492.31 ± 149.75
*p*-Coumaric acid (500 μg/mL)	331.58 ± 11.24	38.15 ± 4.26	6838.46 ± 247.95

The data are shown as means ± SD. ^a,b^ mean values sharing the same superscript letter are not significantly different.

**Table 3:** Selected phytochemical contents in dry ethanolic extracts of propolis [mg/g].

Sample	Caffeic Acid	*p*-Coumaric Acid	Ferulic Acid	Benzoic Acid	CAPE	Vanillin	Pinobanksin	Pinocembrin	Galangin
dEEP1	1.08 ± 0.05 ^a^	24.86 ± 1.24 ^a^	7.66 ± 0.38 ^a^	10.85 ± 0.54 ^a^	6.42 ± 0.32 ^d^	1.61 ± 0.08 ^a^	4.60 ± 0.23 ^b^	4.21 ± 0.21 ^d^	4.17 ± 0.21 ^c^
dEEP2	2.13 ± 0.11 ^c^	45.55 ± 2.28 ^c^	13.16 ± 0.66 ^c^	14.05 ± 0.70 ^c^	3.05 ± 0.15 ^b^	2.59 ± 0.13 ^d^	4.14 ± 0.21 ^b^	1.81 ± 0.09 ^b^	1.10 ± 0.06 ^a^
dEEP3	1.86 ± 0.09 ^b^	39.83 ± 1.99 ^b^	11.33 ± 0.57 ^b^	10.58 ± 0.53 ^b^	0.90 ± 0.04 ^a^	1.92 ± 0.10 ^b^	2.92 ± 0.15 ^a^	0.27 ± 0.01 ^a^	0.92 ± 0.05 ^a^
dEEP4	2.58 ± 0.13 ^d^	46.70 ± 2.33 ^c^	13.52 ± 0.68 ^c^	12.20 ± 0.61 ^b^	4.76 ± 0.24 ^c^	2.28 ± 0.11 ^c^	3.06 ± 0.15 ^a^	2.94 ± 0.15 ^c^	2.79 ± 0.14 ^b^

Data are shown as means ± SD. ^a,b,c,d^—mean values sharing same superscript letter are not significantly different (*p* > 0.05).

**Table 4:** Minimum inhibitory concentrations of tested extracts (EEP1–EEP4) and phenolic standards (*p*-coumaric acid, galangin, and pinocembrin) and antibiotics (chloramphenicol, oxacillin, and streptomycin).

	*Klebsiella pneumoniae*	*Streptococcus agalactiae*
	MIC [µg/mL]	
dEEP1	625.00	78.13
dEEP2	625.00	156.25
dEEP3	NAA	39.06
dEEP4	NAA	39.06
*p*-Coumaric acid	NAA	125.00
Galangin	NAA	NAA
Pinocembrin	31.25	15.62
Chloramphenicol	7.81	1.95
Oxacillin	NAA	31.25
Streptomycin	1.95	0.49

NAA—no antibacterial activity in tested concentration range. (EEP1–EEP4: 0.15–5000 µg/mL; standards: 0.03–500 µg/mL; antibiotics: 0.02–250 µg/mL).

**Table 5:** List of secreted proteins based on keyword search. (Access date: 25 March 2025).

UniProt ID	Function	Link	AlphaFold Model
P64081	Enolase OS = *Streptococcus agalactiae* serotype V (strain ATCC BAA-611/2603 V/R)	https://www.uniprot.org/uniprotkb/P64081	Yes
Q8DX06	Serine protease, subtilase family OS = *Streptococcus agalactiae* serotype V (strain ATCC BAA-611/2603 V/R)	https://www.uniprot.org/uniprotkb/Q8DX06/	No
Q8DYR5	Cell wall surface anchor family protein OS = *Streptococcus agalactiae* serotype V (strain ATCC BAA-611/2603 V/R)	https://www.uniprot.org/uniprotkb/Q8DYR5/	Yes
Q8E1T8	Peptidase OS = *Streptococcus agalactiae* serotype V (strain ATCC BAA-611/2603 V/R)	https://www.uniprot.org/uniprotkb/Q8E1T8/	Yes
Q8DYR9	Cell wall surface anchor family protein OS = *Streptococcus agalactiae* serotype V (strain ATCC BAA-611/2603 V/R)	https://www.uniprot.org/uniprotkb/Q8DYR9/	Yes
Q8E0S5	Cell wall surface anchor family protein, putative OS = *Streptococcus agalactiae* serotype V (strain ATCC BAA-611/2603 V/R)	https://www.uniprot.org/uniprotkb/Q8E0S5/	Yes
A0A0H3GUG9	Enolase OS = *Klebsiella pneumoniae* subsp. pneumoniae	https://www.uniprot.org/uniprotkb/A0A0H3GUG9/	Yes

**Table 6:** The results of the blind docking simulations for the selected proteins and reference ligands. The energy scores are reported for the top-scoring pose for each ligand. The energy values are given in [kcal/mol].

UniProt ID	Score			Energy	
Ligand		Total	Potential	vdW	Electrostatic
A0A0H3GUG9	Enolase OS = *Klebsiella pneumoniae* subsp. Pneumoniae				
galangin	−7.194	23.906	−26.592	−16.127	−10.465
pinocembrin	−7.174	27.963	−25.888	−14.225	−11.663
*p*-coumaric acid	−6.567	−14.429	−27.390	−5.051	−22.339
P64081	Enolase OS = *Streptococcus agalactiae* serotype V (strain ATCC BAA-611/2603 V/R)				
galangin	−7.314	24.453	−28.751	−7.803	−20.948
pinocembrin	−7.291	29.160	−27.769	−10.261	−17.508
*p*-coumaric acid	−6.638	−14.091	−27.657	−4.885	−22.772
Q8E1T8	Peptidase OS = *Streptococcus agalactiae* serotype V (strain ATCC BAA-/2603 V/R)				
galangin	−7.606	32.579	−21.420	−16.787	−4.633
pinocembrin	−7.176	28.490	−21.470	−3.997	−17.473
*p*-coumaric acid	−7.105	−11.272	−23.826	−10.08	−13.746

## Data Availability

All data are contained within the article and Supplementary Material.

## Supplementary Materials

The following supporting information can be downloaded at https://www.mdpi.com/article/10.3390/molecules30092036/s1, Table S1: Correlation matrix for obtained parameters.

## Abbreviations

The following abbreviations are used in this manuscript:

EEP	ethanolic extract of propolis
dEEP	dry ethanolic extract of propolis
FRAP	ferric reducing antioxidant power
DPPH	2,2-Diphenyl-1-picrylhydrazyl
CUPRAC	cupric reducing antioxidant activity
TPC	total phenolic content
TFC	total flavonoid content
MIC	minimum inhibitory concentration
HPLC-DAD	high-performance liquid chromatography with diode array detector
CAPE	caffeic acid phenetyl ester
QE	quercetin equivalents
GAE	gallic acid equivalents
SD	standard deviation
ATCC	American Type Culture Collection
DSM	Deutsche Sammlung von Mikroorganismen
TPTZ	2,4,6-Tris(2-pyridyl)-s-triazine
MHB	Mueller–Hinton broth
MHA	Mueller–Hinton agar
MTT	1-(4,5-Dimethylthiazol-2-yl)-3,5-diphenylformazan
