# Supplementary material for: Selected Polyphenols of Polish Poplar Propolis as a Key Component Shaping Its Antibacterial Properties—In Vitro and In Silico Approaches
Source: Molecules. 2025 May 3;30(9):2036. doi: 10.3390/molecules30092036 (PMC12073398; doi:10.3390/molecules30092036)
Supplement: Supplementary file 1 [file molecules-30-02036-s001.zip › molecules-3597650-supplementary.pdf]

Table S1. Correlation matrix for obtained parameters

	TPC	TFC	FRAP	DPPH	CUPRAC	Caffeic acid	p-Coumaric acid	Ferulic acid	Benzoic acid	Vanillin	Pinobanksin	Pinocembrin	CAPE	Galangin	TFC/TPC	flavonoids/ phenolic acids
TPC	1.000															
TFC	0.712	1.000														
FRAP	0.648	0.360	1.000													
DPPH	0.875	0.959	0.441	1.000												
CUPRAC	0.146	0.564	0.555	0.384	1.000											
Caffeic acid	0.449	-0.300	0.315	-0.018	-0.614	1.000										
p-Coumaric acid	0.250	-0.502	0.313	-0.246	-0.585	0.965	1.000									
Ferulic acid	0.242	-0.509	0.263	-0.248	-0.633	0.970	0.998	1.000								
Benzoic acid	-0.461	-0.874	-0.508	-0.742	-0.894	0.538	0.630	0.661	1.000							
Vanillin	-0.174	-0.800	-0.128	-0.597	-0.808	0.800	0.886	0.901	0.916	1.000						
Pinobanksin	-0.778	-0.248	-0.906	-0.438	-0.165	-0.671	-0.623	-0.589	0.215	-0.191	1.000					
Pinocembrin	-0.045	0.337	-0.740	0.277	-0.292	-0.368	-0.535	-0.483	-0.024	-0.325	0.649	1.000				
CAPE	-0.024	0.345	-0.730	0.291	-0.302	-0.348	-0.519	-0.467	-0.022	-0.317	0.632	1.000	1.000			
Galangin	0.147	0.619	-0.507	0.529	0.008	-0.489	-0.684	-0.645	-0.352	-0.595	0.506	0.943	0.943	1.000		
TFC/TPC	0.371	0.916	0.104	0.768	0.660	-0.653	-0.807	-0.811	-0.891	-0.958	0.118	0.475	0.473	0.737	1.000	
flavonoids/phenolic acids	-0.208	0.437	-0.638	0.255	0.134	-0.757	-0.866	-0.834	-0.331	-0.660	0.757	0.883	0.874	0.921	0.698	1.000

Coefficients marked in red are significant at $p = 0.05$
